# Lethal and Sublethal Effects of Contact Insecticides and Horticultural Oils on the Hibiscus Bud Weevil, *Anthonomus testaceosquamosus* Linell (Coleoptera: Curculionidae)

**DOI:** 10.3390/insects14060544

**Published:** 2023-06-11

**Authors:** A. Daniel Greene, Xiangbing Yang, Yisell Velazquez-Hernandez, German Vargas, Paul E. Kendra, Catharine Mannion, Alexandra M. Revynthi

**Affiliations:** 1 Entomology and Nematology Department, Tropical Research and Education Center, University of Florida, 18905 SW 280th St., Homestead, FL 33031, USA; yvelazquez@ufl.edu (Y.V.-H.); german.vargas@ufl.edu (G.V.); cmannion@ufl.edu (C.M.); 2 Subtropical Horticulture Research Station, United States Department of Agriculture, Agricultural Research Service, Miami, FL 33158, USA; xiangbing.yang@usda.gov (X.Y.); pkendra243@gmail.com (P.E.K.)

**Keywords:** IPM, chemical control, conventional pesticides, biorational pesticides

## Abstract

**Simple Summary:**

The invasive hibiscus bud weevil (HBW), *Anthonomus testaceosquamosus* Linell (Coleoptera: Curculionidae), has presented a challenge to ornamental nurseries in Florida since its arrival in 2017. Hibiscus flowers drop from plants because of HBW feeding and oviposition, which results in plants that are unattractive and subsequently not purchased by consumers. To help growers manage the HBW, we measured the lethal and sublethal effects on HBW adults caused by 21 different insecticide and horticultural oil products. *Laboratory Experiments:* Horticultural oil products only caused mortality when they were directly sprayed on HBW adults. Diflubenzuron, pyrethrins, spinetoram plus sulfoxaflor, and spirotetramat were selected for further analysis in *Contact Toxicity Experiments* and *Greenhouse Experiments* because these products caused either significant mortality and/or a reduction in feeding and oviposition in HBW adults. *Contact Toxicity Experiments*: Pyrethrins, spinetoram plus sulfoxaflor, and spirotetramat were highly toxic to HBW adults. *Greenhouse Experiments:* Pyrethrins application significantly reduced HBW adult feeding/oviposition on, and the number of larvae within, hibiscus buds. We recommend that HBW infestations be managed with rotations of diflubenzuron, pyrethrins, spinetoram plus sulfoxaflor, spirotetramat, and horticultural oils, as the rotation of products belonging to different insecticide groups can reduce the risk of resistance development while still providing control.

**Abstract:**

In 2017, the hibiscus bud weevil (HBW), *Anthonomus testaceosquamosus* Linell (Coleoptera: Curculionidae), was found outside of its native range of Mexico and Texas, infesting hibiscus plants in Florida. Therefore, we selected 21 different insecticide and horticultural oil products to evaluate their effects on the reproductive rate, feeding, and oviposition behavior of the HBW. In laboratory experiments, significant mortality was observed in adult weevils exposed to diflubenzuron-treated hibiscus leaves and buds, and hibiscus buds treated with diflubenzuron contained the fewest number of eggs and feeding/oviposition holes. Among horticultural oil products, significant mortality was only observed in experiments in which adult weevils were directly sprayed (direct experiments). Pyrethrins and spinetoram plus sulfoxaflor reduced the oviposition rate and caused significant mortality in direct experiments. Diflubenzuron, pyrethrins, spinetoram plus sulfoxaflor, and spirotetramat were further tested via contact toxicity experiments and greenhouse experiments. Contact toxicity experiments demonstrated that the tested insecticides (except diflubenzuron) were highly toxic to HBW adults. In greenhouse experiments, only those hibiscus plants treated with pyrethrins had significantly fewer feeding/oviposition holes and larvae within their flower buds when compared to control (water-treated) plants. These results constitute an important first step in the identification of effective chemical control options for the HBW.

## 1. Introduction

In its native range in northeastern Mexico and southern Texas, the hibiscus bud weevil (HBW), *Anthonomus testaceosquamosus* Linell (Coleoptera: Curculionidae), has been associated with multiple malvaceous host plants, including *Abutilon abutiloides* (Jacq.) Garcke ex Hochr., *Allowissadula lozanii* (Rose) R. E. Fries, *Malvastrum spicatum* (L.) A. Gray, and China rose hibiscus, *Hibiscus rosa-sinensis* L. [1,2,3,4]. However, in 2017, the HBW was discovered feeding on China rose hibiscus (hereafter “hibiscus”) in Miami-Dade County, Florida [3]. In addition to feeding on hibiscus flower buds, HBW females also oviposit within them. Larvae feed on pollen within hibiscus buds and develop through three larval instars and a pupal stage before adult weevils emerge [2,5]. Feeding- and oviposition-related damage to hibiscus buds can also cause bud abortion in plants, thereby resulting in unmarketable plants lacking flowers [2].

The detection of HBW in Miami-Dade County is particularly problematic due to the size of the floriculture industry in south Florida. In 2019, 70% (7 million plants) of the hibiscus plants sold in the U.S. were produced in Florida [6]. Miami-Dade County’s contribution to Florida’s hibiscus industry is significant, as it ranks 1st among all U.S. counties for market value of nursery, greenhouse, floriculture, and sod commodities sold [7]. Recognizing the significance of the threat posed by the HBW and preventing its spread to other hibiscus-producing states, the Florida Department of Agriculture and Consumer Services-Division of Plant Industry (FDACS-DPI) regulates this pest. A compliance agreement is issued by FDACS-DPI to any nursery in which HBW has been detected. The agreement stipulates the protocol for hibiscus scouting, record-keeping, and insecticide applications and allows growers to ship nursery products out of nurseries if there is no indication of HBW presence on the plants. 

Per the compliance agreement, contact insecticides approved for weevil control in greenhouse and/or nursery settings are required to be applied every 7–10 days during bud development. Given that studies on HBW biology and ecology have only recently begun (see [5]), trap-based monitoring and effective biological control tactics have yet to be developed. Insecticide applications remain one of the only methods available for controlling this pest. While insecticides belonging to the pyrethroid (3A) and neonicotinoid (4A) groups have been found to cause significant mortality in the congeneric pepper weevil, *Anthonomus eugenii* Cano [8,9], and cotton boll weevil, *A. grandis* Boheman [10], resistance to pyrethroid insecticides has been reported for these pests [11,12]. Although management efforts specifically targeting the HBW have only recently begun in south Florida, the threat of insecticide resistance is an important factor to consider, particularly given the biological traits that this pest possesses. Specifically, the HBW can complete its life cycle within 16 days at 27 °C [5]. While the potential for the occurrence of multiple HBW generations per year in southern Florida agroecosystems is troublesome on its own for hibiscus nurseries operating in the area, the threat of resistance is increased for pests that complete multiple generations per year when compared with those that complete one or fewer [13].

To better manage the HBW, the adoption of insecticides with different modes of action within the pest management program will not only provide effective control but will also lower the selection pressure for insecticide resistance development [13]. Within ornamental markets, insecticide rotation with neonicotinoids for pest control is no longer an option, as clientele demand ornamental commodities that have not been treated with these products due to the detrimental effects that have been reported for pollinator exposure to these insecticides [14,15,16]. Therefore, the identification of other insecticide products with reduced non-target effects that can be used in rotation for the management of the HBW is a priority. Horticultural oils (i.e., plant-derived essential oils or highly refined petroleum oils [including mineral oils]) fulfill this requirement, as natural enemies face little risk if direct contact is not made between the product and the arthropod [17]. When applied to plants, horticultural oils can repel insects, and virus transmission can be prevented by feeding behavior modification. Mortality can occur through asphyxiation via spiracle blockage or poisoning via metabolic process alteration [18,19,20]. Horticultural oils have also been previously used in weevil management, as oil application to citrus leaves successfully exposed Citrus root weevil, *Diaprepes abbreviatus* L., eggs that had been previously hidden from natural enemies and unfavorable environmental conditions [21].

Due to the designation of the HBW as a regulated pest, hibiscus operations require effective chemical products that can provide an instantaneous effect on HBW infestations to be able to ship their plants. Additionally, the adult stage is the only developmental stage of the HBW that is found outside of the hibiscus buds, as larval and pupal stages benefit from the protection provided by development within hibiscus buds. Therefore, our study focused on measuring the effect of contact insecticides on HBW adults, as these formulations can affect insects via direct contact immediately after application [22]. Specifically, we chose to measure the lethal and sublethal (e.g., feeding and oviposition behavior and reproductive rate) effects of 21 different commercially available insecticides and horticultural oils representing 12 different insecticide groups against HBW adults via contact application in laboratory and greenhouse studies.

## 2. Materials and Methods

### 2.1. Hibiscus Plants

Potted hibiscus plants (var. “Painted lady”) used for experiments and to sustain the HBW colony were grown outdoors. Plants were sourced from local nurseries and fertilized every three months with slow-release fertilizer (8-2-12: N-P-K) (Diamond R Fertilizer, Fort Pierce, FL, USA). Overhead irrigation was provided twice a day.

### 2.2. Hibiscus Bud Weevil Colony

Hibiscus buds infested with immature HBW stages were stored in plastic food storage containers and maintained in Percival I-36LL incubators at 27 ± 1 °C, 12:12 h L:D, and 60% RH (Percival Geneva Scientific, Williams Bay, WI, USA) until adult emergence. Adult weevils emerging from these hibiscus buds were placed inside 30.5 × 30.5 × 30.5 cm mesh cages (BioQuip^®^ Products, Rancho Dominguez, CA, USA) alongside fresh hibiscus buds for them to feed on and oviposit within; mesh cages were also maintained within Percival I-36LL incubators at 27 ± 1 °C, 12:12 h L:D, and 60% RH. The colony was occasionally supplemented with weevils collected from local nurseries. All weevils (≈1 week old) used for experimentation were provided by the colony.

### 2.3. Laboratory Bioassays

**Figure 1:**
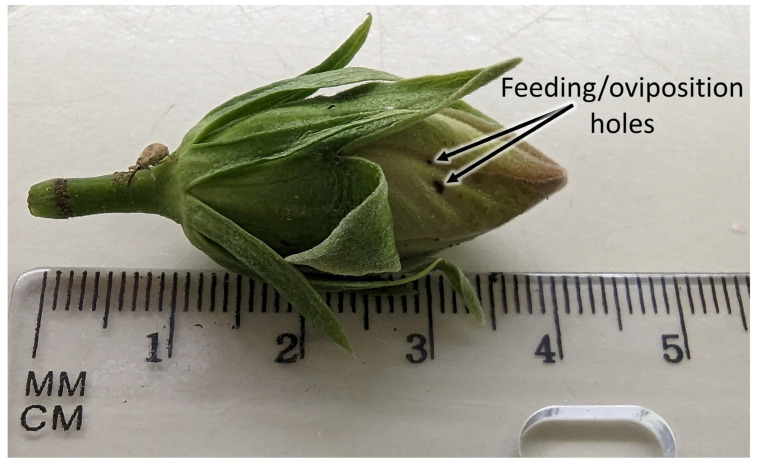
“Painted lady” variety hibiscus flower bud displaying damage (holes) from hibiscus bud weevil feeding and/or oviposition.

Insecticide and horticultural oil products used in experiments were selected based on being either recommended by the FDACS-DPI compliance agreement, used for control of Coleopteran/Curculionid pests, or approved for use on ornamental plants in nurseries (Table 1). One-liter experimental solutions were created by combining each product at the recommended label rate (Table 1) with water in a 1.7 L handheld sprayer; control solutions contained only water. A group of 3–6 “Painted Lady” variety hibiscus plants were then sprayed outdoors with a given insecticide, oil, or water solution and allowed to dry for 4 h. After 4 h, 20 flower buds and 10 leaves were collected from each plant in each treatment group. Sprayed plants used in experiments were maintained outdoors on irrigated plant pads for the duration of the experiments. Flower buds and leaves collected from sprayed plants were used to establish two types of experiments immediately after collection: fixed and replacement experiments. In fixed experiments, a single sprayed flower bud or leaf was added to a petri dish (Corning™ 100 × 15 mm, Fisher Scientific, Pittsburgh, PA, USA) containing two HBW adults (one male and one female). Weevil mortality was assessed at 24-, 48-, 72-, and 96-hours post-experimental setup (PES checkpoint). Replacement experiments were set up in the same manner as fixed experiments, but at each PES checkpoint, the flower bud within each petri dish was replaced with a freshly picked flower bud from previously sprayed plants. Flower buds removed at each PES checkpoint were dissected, and the number of feeding holes and oviposited eggs were counted (Figure 1). Direct spray experiments (hereafter “direct experiments”) were established by applying 1 mL (volume released by a single spray pump of a micropipette) of a given insecticide or oil solution directly onto a single HBW adult within a petri dish. Although the entire 1 mL solution was pipetted directly onto each weevil, petri dishes contained 3 filter papers (9 cm diameter, P5, Fisher Scientific, Pittsburgh, PA, USA) to absorb excess solution. Weevil mortality was assessed at 24-, 48-, 72-, and 96-h PES. Each laboratory experiment (fixed, replacement, and direct) included a water control administered using the same methodology as the treatment solutions, and each experiment was replicated 10 times (a petri dish = an experimental unit).

Based on the results of laboratory experiments, the four most effective insecticide products that resulted in either the highest mortality and/or the lowest feeding- or oviposition-related damage (diflubenzuron, pyrethrins, spinetoram plus sulfoxaflor, and spirotetramat) were further screened via contact toxicity experiments and greenhouse experiments to determine their effects on HBW mortality, feeding behavior, oviposition, and development.

### 2.4. Contact Toxicity Experiments

To understand the dose-dependent toxicity of the four effective insecticides identified in laboratory experiments, topical bioassays (via thoracic application) were conducted to determine the median lethal dose of pyrethrins, spinetoram plus sulfoxaflor, spirotetramat, and diflubenzuron on HBW adults under laboratory conditions at 26.0 ± 1.0 °C, 70.0 ± 5.0% RH, and 12:12 L:D photoperiod in the Insect Toxicology Laboratory at the USDA-ARS facility in Miami, Florida. One-liter stock solutions were created by combining the liquid solutions of pyrethrins or spirotetramat, the water-dispersible granules of spinetoram plus sulfoxaflor, or the wettable powder of diflubenzuron with reverse osmosis (RO) water at a rate of 49.1 g/L for pyrethrins, 240 g/L for spirotetramat, the max label rate of 0.26 g/L for spinetoram plus sulfoxaflor, and the max label rate of 1.2 g/L for diflubenzuron. Serial dilutions of each stock solution were made with RO water at concentrations of 0.02, 0.04, 3.06, 6.13, and 12.25 µg/µL for pyrethrins; 20, 40, 80, and 120 µg/µL for spirotetramat; 0.0325, 0.065, 0.13, and 0.26 µg/µL for spinetoram plus sulfoxaflor; and 0.3, 0.6, and 1.2 µg/µL for diflubenzuron. Each concentration of each insecticide was evaluated for its toxicity against HBW adults.

To conduct topical bioassays, HBW adults (≈1 week old) were first placed in plastic petri dishes (12 cm in diameter × 3 cm in height) of ≈10 females or up to 25 males per dish (replicate). Adults were then anesthetized at 4 °C in a refrigerator for 5 min to facilitate the topical application. A repeating dispenser equipped with a gastight microliter syringe (50 µL) (PB600, Hamilton Company, Reno, NV, USA) was used to apply 1 µL of each insecticide concentration (or water in control replicates) to the dorsal thorax of an HBW adult. After topical application, sugar and water agar were added to petri dishes. After 24 h, the numbers of live and dead HBW adults were documented, and HBW mortality was calculated. Moribund adults capable of limb movement but unable to crawl were considered dead. Adults treated with RO water along with each insecticide were used as controls. For each dilution, around 10 female adult HBW were treated, and up to 25 male adult HBW were treated. Each treatment was replicated 3 times, and a total of 479 females and 500 males were used in the evaluation of all insecticide treatments.

### 2.5. Greenhouse Experiments

Insect-proof (61 × 61 × 91 cm: L × W × H) 100 μm mesh cages (BioQuip^®^ Products, Rancho Dominguez, CA, USA) (30 total cages) containing a single “Painted Lady” variety hibiscus plant were individually infested with two pairs of HBW adults (two males and two females) under greenhouse conditions (27 °C, 70% ± 10 RH, including drip irrigation). Before insecticide solutions were applied to plants, a baseline level of infestation was recorded four days post-infestation (PES) by counting the number of HBW eggs, larvae, and holes (feeding and/or oviposition) within five randomly selected flower buds taken from each plant. One week after weevils were introduced (7 days PES), a treatment of either diflubenzuron, pyrethrins, spinetoram plus sulfoxaflor, spirotetramat, or water (control) was randomly applied to each hibiscus plant (6 plants [replicates] per treatment). Treatment solutions were created following the same protocol used in laboratory experiments, and plants were sprayed until runoff within mesh cages. Each plant was sprayed again at 19 days PES to target weevils that were developing inside hibiscus buds during the first spray. At each checkpoint of 1-, 4-, 7-, 19-, and 28-days PES, five flower buds (or as many as possible if a plant had <5) were collected from each hibiscus plant, and the level of weevil infestation was assessed via enumeration of the number of HBW eggs, larvae, and holes (feeding and/or oviposition) within each bud. 

### 2.6. Statistical Analyses

#### 2.6.1. Mortality Measurements

The effect of insecticides and horticultural oils on HBW mortality in laboratory experiments was assessed via logistic regression. A separate model was constructed for each experiment type (i.e., fixed experiments: leaf, fixed experiments: flower bud, replacement experiments: flower bud, and direct experiments). For each analysis, model convergence was achieved by including only those treatments in which mortality was observed during at least one PES checkpoint and only those PES checkpoints in which mortality was observed in at least one treatment. Each model tested the categorical fixed effects of horticultural oil and insecticide products and PES checkpoint on the proportion of dead weevils (out of a total of 2; response variable) in each petri dish. The effect of temporal pseudoreplication associated with the collection of mortality data from each petri dish at multiple PES checkpoints was incorporated into models via the inclusion of the random effect of variable model intercepts for each petri dish (hereafter “ID”). For each regression model, ANOVA was used to determine whether the mean proportion of dead weevils differed from the grand mean proportion of dead weevils for at least one insecticide and horticultural oil product (across time) or for at least one PES checkpoint (across treatments). Pairwise comparisons among insecticide and horticultural oil products and PES checkpoints were conducted for each regression model. 

#### 2.6.2. Biological Measurements

The number of feeding holes and eggs per flower bud in replacement experiments, along with the number of feeding holes, eggs, and the total number of larvae (number dead plus the number alive, hereafter “larvae”) per flower bud in greenhouse experiments, were separately analyzed via generalized linear mixed modeling with a negative binomial error structure if response variables had a variance: mean ratio of >3 [23]. For response variables in which the variance: mean ratio was <3, both poisson and negative binomial models were constructed, and the difference in model fit was assessed via likelihood ratio test. Each model included insecticide andhorticultural oil products and PES checkpoint as the categorical fixed effects. An intercept that varied among replicates and among each replaced bud within replicates was used for the random effect component of models constructed for replacement experiment data (the number of feeding holes and eggs per flower bud). Models constructed for greenhouse experiment data included the same random effect structure used in mortality measurement models to account for the effect of temporal pseudoreplication. For each regression model, ANOVA was used to determine whether the mean number of feeding holes, eggs, or larvae differed from the grand mean number of feeding holes, eggs, or larvae for at least one treatment (across time) or for at least one PES checkpoint (across treatments). Pairwise comparisons among treatments and PES checkpoints were conducted for each regression model. 

Familywise error rates were not adjusted for pairwise comparisons in this study, as we chose to minimize the probability of making a Type II error (i.e., failing to reject a null hypothesis of no effect for an insecticide and horticultural oil product). This decision reflects the urgent need to identify effective chemical treatments for HBW that can be used in rotation in nursery settings.

#### 2.6.3. Contact Insecticide Toxicity Analysis

Mortality data from HBW males and females in topical bioassays across each concentration was used to calculate the median lethal dose (LD_50_) for each insecticide. Prior to analysis, mortality data for each treatment were corrected with mortality data in control replicates using Abbott’s formula [24]. A probit analysis was then used to calculate the lethal dose corresponding to a 50% reduction (LD_50_) in male or female survival based on the regression curve. Analyses were performed using SAS version 9.4 (SAS Institute, Cary, NC, USA, 2020).

#### 2.6.4. Software

All analyses were conducted in R (v4.2.0) [25], except for the contact toxicity analysis. Regression analyses were completed using the *glmmTMB* package (v1.1.3) [26], while post hoc analyses used the *emmeans* (v1.7.3) [27] and *multcomp* packages (v1.4.18) [28]. Graphical data displays were produced with the *ggplot2* package (v3.3.5) [29].

## 3. Results

### 3.1. Laboratory Experiments

#### 3.1.1. Fixed Experiments

##### Hibiscus Bud Experiments: Mortality

**Figure 2:**
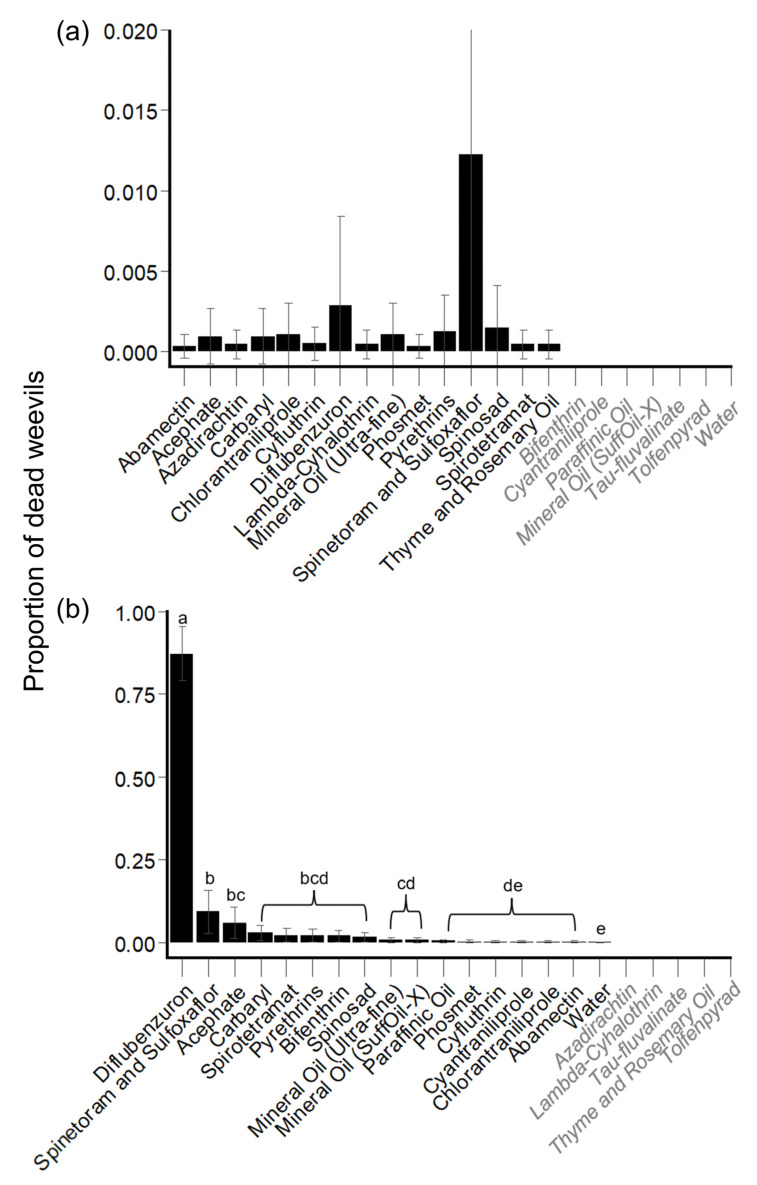
Generalized linear mixed model (GLMM)-predicted proportion of HBW mortality (±SE) and observed HBW mortality (±SE) among insecticide and horticultural oil products across all post-experimental setup (PES) checkpoints. (**a**) Fixed experiments: hibiscus bud; and (**b**) Fixed experiments: hibiscus leaf. Black = modeled treatments; GLMM-predicted proportion of mortality. Grey and italicized = treatments not included in models due to causing 0 or 100 percent mortality (model convergence); observed mortality data. Treatments with different letters were significantly different from one another (*p* < 0.05; *t*-tests).

Across all treatments, all weevils survived through the first checkpoint (24-h PES), and the mortality of weevils significantly increased at each PES checkpoint in which mortality was observed (48-, 72-, and 96-h PES; Wald *X*^2^ test: *X*^2^ = 21.5, df = 2, *p* < 0.001). There were no significant differences among insecticide and horticultural oil products on the proportion of dead weevils in hibiscus bud experiments, however (Wald *X*^2^ test: *X*^2^ = 4.52, df = 14, *p* = 0.99) (Figure 2a, Supplementary Figure S1).

##### Hibiscus Leaf Experiments: Mortality

Across all treatments, weevil mortality significantly increased at each PES checkpoint (24-, 48-, 72-, and 96-h PES; Wald *X*^2^ test: *X*^2^ = 56.04, df = 3, *p* < 0.001). Across all PES checkpoints, diflubenzuron caused significantly higher mortality (0.87 Estimated Marginal Mean of the proportion of dead weevils [EMM] [0.62–0.97 95% CI]) than all other insecticide and horticultural oil products, including water control. Additionally, spinetoram plus sulfoxaflor (0.09 EMM [0.02–0.31 95% CI]) and acephate (0.06 EMM [0.01–0.25 95% CI]) caused significantly higher HBW mortality than paraffinic oil, phosmet, cyfluthrin, cyantraniliprole, chlorantraniliprole, abamectin, and water control (Figure 2b, Supplementary Figure S2).

#### 3.1.2. Direct Mortality

The model containing HBW gender as a fixed effect (in addition to the fixed effects of (1) insecticide and horticultural oil product and (2) PES checkpoint and random intercepts for ID) did not provide a better fit to the data (likelihood ratio test: *X*^2^ = 0.36, df = 1, *p* = 0.55) than the reduced model (containing the fixed effects of only (1) insecticide and horticultural oil product and (2) PES checkpoint and random intercepts for ID). Therefore, the reduced model was selected as the final model. While each horticultural oil product (mineral oils of suffoil-X or ultra-fine, paraffinic oil, and thyme and rosemary oil) caused 100% mortality in each replicate by the first PES checkpoint (24 h), these treatments were not included in the final model to allow for model convergence.

**Figure 3:**
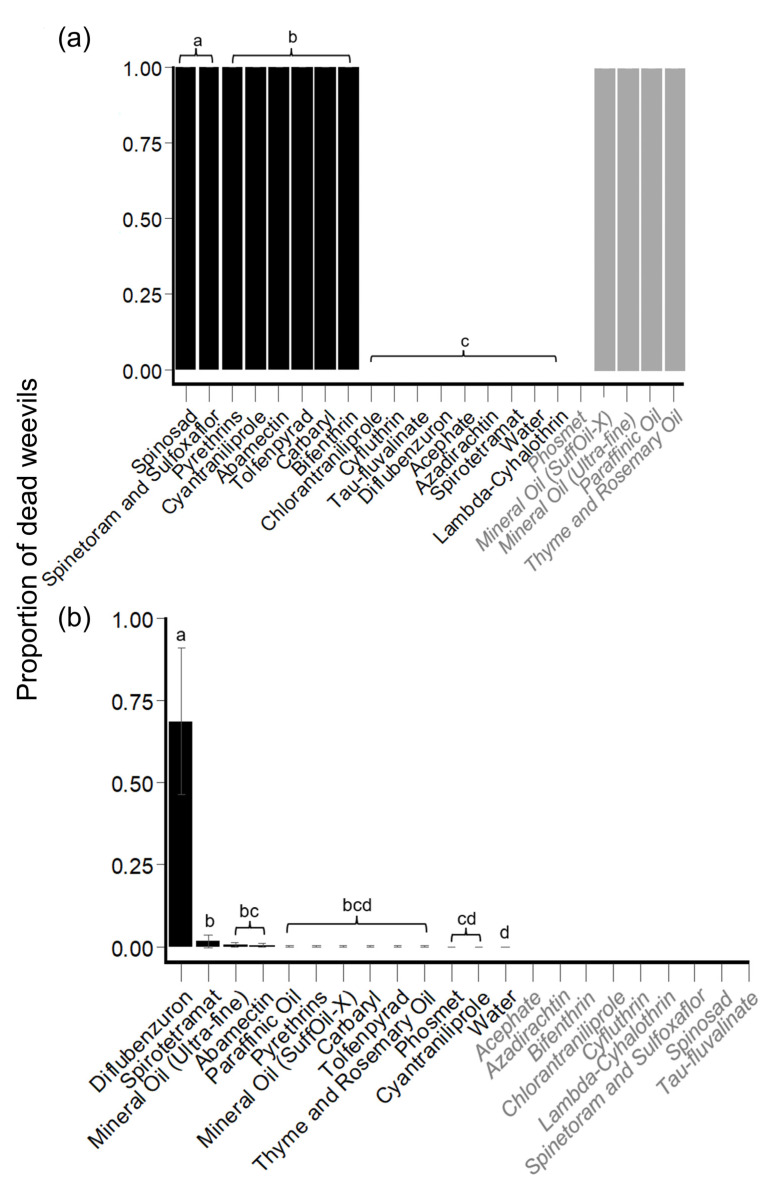
Generalized linear mixed model (GLMM)-predicted proportion of HBW mortality (±SE) and observed HBW mortality (±SE) among insecticide and horticultural oil products across all post-experimental setup (PES) checkpoints. (**a**) Direct experiments; and (**b**) replacement experiments: hibiscus bud. Black = modeled treatments; GLMM-predicted proportion of mortality. Grey and italicized = treatments not included in models due to causing 0 or 100 percent mortality (model convergence); observed mortality data. Treatments with different letters were significantly different from one another (*p* < 0.05; *t*-tests).

Across all treatments, weevil mortality significantly increased (Wald *X*^2^ test: *X*^2^ = 107, df = 3, *p* < 0.001) at each PES checkpoint until it reached 0.99 EMM [0.997–0.999 95% CI] at 96 h PES. Across all PES checkpoints, HBW mortality caused by spinosad (1.000 EMM [1.000–1.000 95% CI]) and spinetoram plus sulfoxaflor (1.000 EMM [1.000–1.000 95% CI]) was significantly higher than the mortality for all other insecticide and horticultural oil products (including water control). Pyrethrins, cyantraniliprole, abamectin, tolfenpyrad, carbaryl, and bifenthrin resulted in significantly higher mortality (EMMs of 0.99) than chlorantraniliprole, cyfluthrin, tau-fluvalinate, diflubenzuron, acephate, spirotetramat, azadirachtin, water control, and lambda-cyhalothrin (EMMs of 0) (Figure 3a, Supplementary Figure S3).

#### 3.1.3. Replacement Experiments

##### Hibiscus Bud Experiments: Mortality

Across all treatments, weevil mortality significantly increased at each time point except the period between 48 h and 72 h PES (*t*-test: *t* ratio = −1.1, df = 703, *p* = 0.27). Across all PES checkpoints, diflubenzuron caused significantly higher mortality (0.69 EMM [0.22–0.95 95% CI]) than all other insecticide and horticultural oil products (including water control), while mortality observed for spirotetramat (0.02 EMM [0.001–0.17 95% CI]) was significantly higher than the mortality for phosmet, cyantraniliprole, and water control (Figure 3b, Supplementary Figure S4).

##### Hibiscus Bud Experiments: HBW Eggs

**Figure 4:**
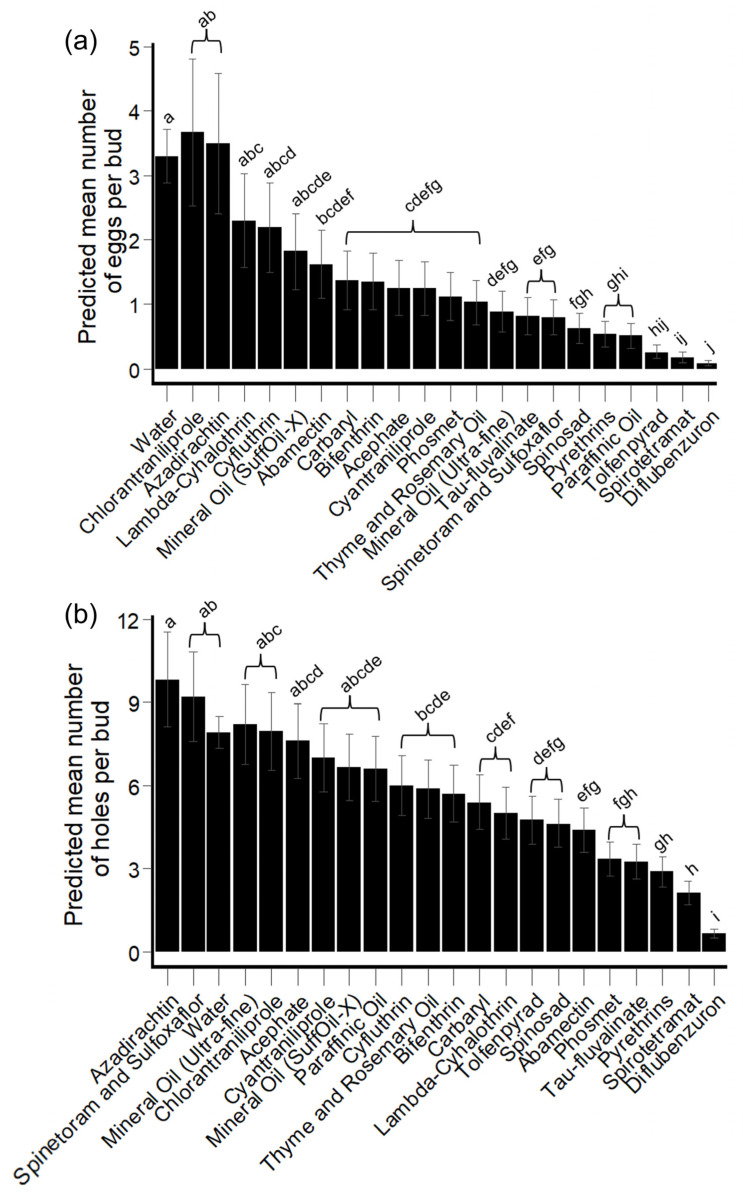
Generalized linear mixed model (GLMM)-predicted mean number (±SE) of HBW eggs and feeding/oviposition holes per hibiscus bud among insecticide and horticultural oil products across all post-experimental setup (PES) checkpoints. (**a**) Replacement experiments: number of HBW eggs within each collected hibiscus bud; and (**b**) replacement experiments: number of HBW feeding/oviposition holes within each collected hibiscus bud. Treatments with different letters were significantly different from one another (*p* < 0.05; *t*-tests).

Across all treatments, the number of HBW eggs per bud significantly decreased at each time point except for the period between 48 h and 72 h PES (*t*-test: *t* ratio = 1.04, df = 1052, *p* =0.63). Across all PES checkpoints, hibiscus buds treated with diflubenzuron, spirotetramat, and tolfenpyrad contained fewer eggs per bud than any treatment (EMM of < 0.3 eggs per bud), while hibiscus buds treated with azadirachtin, chlorantraniliprole, and water control contained an EMM of at least 3 eggs per bud (Figure 4a, Supplementary Figure S5).

##### Hibiscus Bud Experiments: HBW Feeding/Oviposition Holes

Across all treatments, hibiscus buds removed at 24 h PES contained more HBW feeding/oviposition holes than at any other PES checkpoint—significantly more HBW feeding/oviposition holes than the number found within hibiscus buds removed at 48 h (*t*-test: *t* ratio = 8.4, df = 1301, *p* < 0.001), 72 h- (*t*-test: *t* ratio = 9.4, df = 1301, *p* < 0.001), and 96 h PES (*t*-test: *t* ratio = 9.7, df = 1301, *p* < 0.001). Across all PES checkpoints, hibiscus buds treated with diflubenzuron contained significantly fewer HBW feeding/oviposition holes (0.65 EMM [0.4–1.07 95% CI]) per bud than any other treatment, while buds treated with azadirachtin had the highest number of feeding/oviposition holes (9.82 EMM [6.97–13.84 95% CI]) (Figure 4b, Supplementary Figure S6).

#### 3.1.4. Contact Insecticide Toxicity

The dose-dependent experiment demonstrated that the tested insecticides, with the exception of diflubenzuron, were highly toxic to HBW adults (Table 2). Overall, the median lethal doses (LD_50_) of each insecticide were not significantly different between male and female weevils (based on 95% CI comparisons), despite the fact that female LD_50_ doses were higher than LD_50_ doses for males for each insecticide. For HBW males, 0.595, 0.0695, and 41.72 µg per weevil of spinetoram and sulfoxaflor, pyrethrins, and spirotetratmat, respectively, was required to achieve 50% mortality, whereas for females, 50% mortality required 0.061, 0.0955, and 44.36 µg per weevil of spinetoram and sulfoxaflor, pyrethrins, and spirotetratmat, respectively (Table 2). Because weevil mortality was not observed 24 h after the application of the stock solution of diflubenzuron (1.2 g/L), the toxicity of this product was unable to be tested.

### 3.2. Greenhouse Experiments

#### 3.2.1. Collected Hibiscus Buds: HBW Eggs

Because the variance: mean ratio of the number of HBW eggs was <3 (2.2), both poisson and negative binomial models were constructed to assess the effects of insecticide product and PES checkpoint on the number of HBW eggs contained within hibiscus buds in greenhouse experiments. As the negative binomial model provided a significantly better fit (likelihood ratio test: *X*^2^ = 9.9, df = 1, *p* = 0.002) to the data than the poisson model, the former was chosen as the final model.

**Figure 5:**
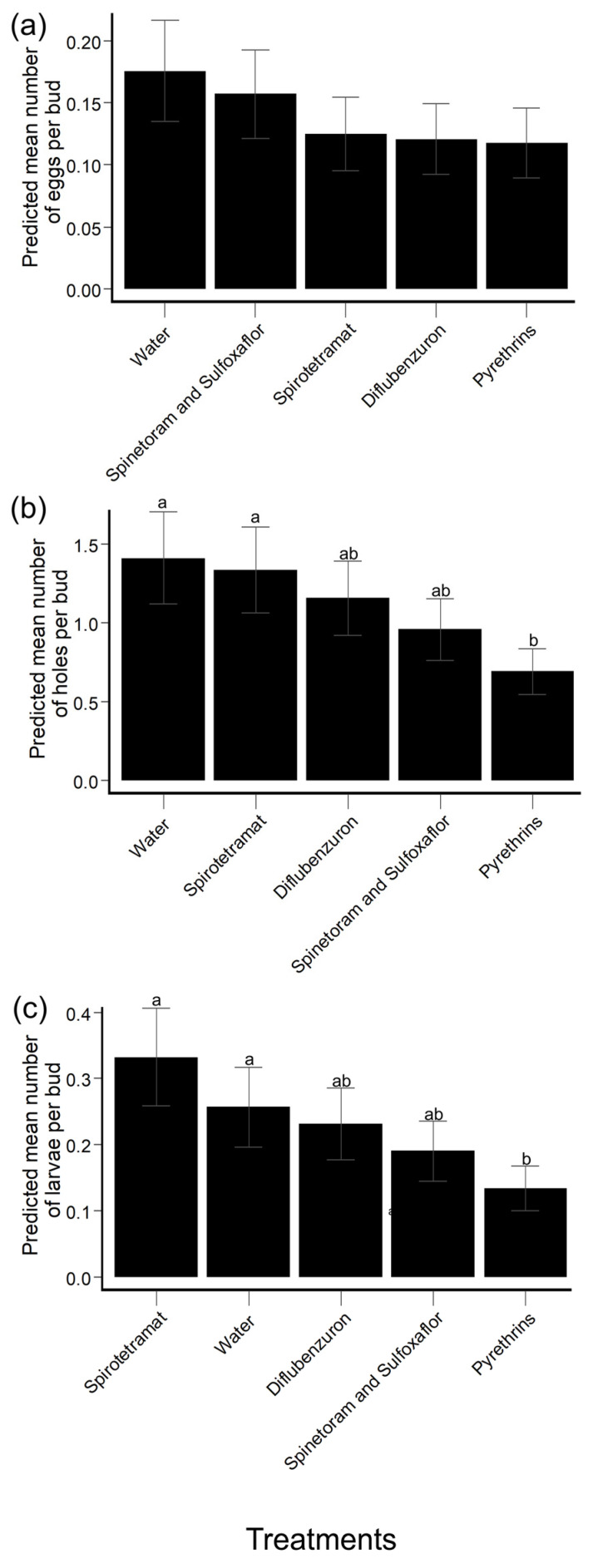
Generalized linear mixed model (GLMM)-predicted mean number (±SE) of HBW eggs, feeding/oviposition holes, and larvae per hibiscus bud among insecticide products and water control across all post-experimental setup (PES) checkpoints in greenhouse experiments. (**a**) Number of HBW eggs; (**b**) number of HBW feeding/oviposition holes; and (**c**) number of HBW larvae within each collected hibiscus bud. Treatments with different letters were significantly different from one another (*p* < 0.05; *t*-tests).

Across all treatments, hibiscus buds collected at 28 days PES contained fewer HBW eggs than at any other PES checkpoint—significantly fewer HBW eggs than the number found within hibiscus buds collected at 19 days (*t*-test: *t* ratio = 3.2, df = 805, *p* = 0.01), 1 day (*t*-test: *t* ratio = 3.6, df = 805, *p* = 0.003), and 0 days PES (*t*-test: *t* ratio = 8.4, df = 805, *p* < 0.0001). There were no significant differences in the number of HBW eggs in collected hibiscus buds among treatments, however (Wald *X*^2^ test: *X*^2^ = 3.01, df = 4, *p* = 0.56) (Figure 5a, Supplementary Figure S7).

#### 3.2.2. Collected Hibiscus Buds: HBW Feeding/Oviposition Holes

Across all treatments, hibiscus buds collected at 7 days PES (0.32 EMM, [0.21–0.49 95% CI]) contained significantly fewer HBW feeding/oviposition holes than those from any other PES checkpoint. Across all PES checkpoints, hibiscus buds collected from pyrethrins-treated plants (0.69 EMM [0.46–1.04 95% CI]) contained significantly fewer feeding/oviposition holes than those collected from water-treated plants (*t*-test: *t* ratio = −2.45, df = 805, *p* = 0.01) and spirotetramat-treated plants (*t*-test: *t* ratio = −2.3, df = 805, *p* = 0.02) (Figure 5b, Supplementary Figure S8).

#### 3.2.3. Collected Hibiscus Buds: HBW Larvae

Because the variance: mean ratio of the number of HBW eggs was <3 (2.1), both poisson and negative binomial models were constructed to assess the effects of insecticide product and PES checkpoint on the number of HBW larvae contained within hibiscus buds in greenhouse experiments. As the negative binomial model provided a significantly better fit (likelihood ratio test: *X*^2^ = 14.9, df = 1, *p* <0.001) to the data than the poisson model, the former was chosen as the final model.

Across all treatments, hibiscus buds collected at 28 days (0.06 EMM [0.03–0.12 95% CI]), 19 days (0.09 EMM [0.05–0.15 95% CI]), and 7 days PES (0.09 EMM [0.05–0.18 95% CI]) contained significantly fewer HBW larvae than hibiscus buds collected at 4 days (0.31 EMM [0.22–0.45 95% CI]), 0 days (0.57 EMM [0.43–0.75 95% CI]), and 1 day PES (1.2 EMM [0.95–1.5 95% CI]). Across all PES checkpoints, hibiscus buds collected from pyrethrins-treated plants (0.13 EMM [0.08–0.22 95% CI]) contained significantly fewer HBW larvae than hibiscus buds collected from water control plants (*t*-test: *t* ratio = −2, df = 805, *p* = 0.045) and spirotetramat-treated plants (*t*-test: *t* ratio = −2.9, df = 805, *p* = 0.004) (Figure 5c, Supplementary Figure S9).

## 4. Discussion

In this study, we sought to test 21 different insecticide and horticultural oil products for their effect on HBW mortality, behavior, and oviposition in laboratory and greenhouse experiments, and the contact toxicity of weevils to the four most effective insecticides was further screened. Twelve different IRAC groups were represented among the 21 tested products, including the mechanical and physical disruption of the mineral and paraffinic oil products, and the repellant, suffocant, endocrine disruption, and neurotransmitter interference properties of thyme and rosemary oil. While various insecticide and horticultural oil products were selected for inclusion in this study, neonicotinoids (IRAC Group 4A), a group of insecticides capable of providing efficient control of ornamental insect pests [15,16], were not. The lack of inclusion of neonicotinoid products in this study reflects current trends among consumers and ornamental markets, as these entities demand plants from nurseries that have not been treated with neonicotinoid products [15].

In laboratory bioassays, diflubenzuron had the greatest overall effect on weevils, as the EMM proportion of dead weevils in diflubenzuron-treated hibiscus leaves and buds reached 0.87 and 0.69 in hibiscus leaf and replacement experiments, respectively. Furthermore, buds treated with diflubenzuron in replacement experiments contained the fewest number of eggs and feeding/oviposition holes. The diflubenzuron-related reduction in the reproductive ability of the HBW that we observed has also been reported for several other insect orders (Diptera, Orthoptera, and Lepidoptera) [30,31,32] and coleopteran families (Coccinellidae and Scarabaeidae) [33,34], including Curculionidae. Previous research found the ability of diflubenzuron to be effective enough in its control of the congeneric cotton boll weevil to warrant its usage in the eradication trials for this pest in North Carolina and Virginia in 1979 [35]. Furthermore, diflubenzuron has demonstrated the capacity to sterilize both male and female cotton boll weevils when combined with an irradiation source and to reduce egg hatch and larval development [35]. When applied to cotton plants under field conditions, diflubenzuron was found to reduce the cuticle hardness, hatch, and flight activity of cotton boll weevil adults that were allowed to feed on treated plants [36]. In contrast with the HBW mortality that we observed following diflubenzuron application to hibiscus leaves and buds, HBW mortality was not observed for this product in direct experiments or in contact toxicity experiments, even when applied to weevils at the max label rate of 1.2 g/L. These data suggest that the ingestion of diflubenzuron plays a significant role in its ability to cause mortality in insects. This observation is supported by De Clercq et al. [37], as the authors discovered that while direct and residual contact with diflubenzuron was harmless to spined solider bugs, *Podisus maculiventris* Say (Hemiptera: Pentatomidae), significant nymphal mortality was observed when the product was ingested with drinking water.

Pyrethrins, spinetoram, sulfoxaflor, and spirotetramat altered weevil behavior and/or reproduction in laboratory experiments as well, as hibiscus buds sprayed with these products were found to have an EMM of fewer than one egg per bud in replacement experiments. Additionally, hibiscus buds treated with pyrethrins and spirotetramat had significantly fewer holes than the water control in replacement experiments. Given the efficacy of diflubenzuron, pyrethrins, spinetoram plus sulfoxaflor, and spirotetramat in laboratory bioassays, greenhouse experiments using these products revealed that only those hibiscus plants treated with pyrethrins had significantly fewer holes and larvae within their flower buds when compared to control (water) plants. No insecticide product significantly reduced the number of eggs found within hibiscus flower buds to a level below that of hibiscus buds from water control plants.

Laboratory toxicity experiments demonstrated that pyrethrins and spinetoram plus sulfoxaflor were highly toxic to HBW males and females, as <0.1 µg per weevil caused 50% mortality in both sexes, while 99% mortality was predicted to occur with an application of and <20.0 µg per weevil. Although pyrethrins and spinetoram plus sulfoxaflor were unable to cause sufficient mortality in hibiscus leaf or bud (fixed and replacement) experiments, the EMM proportion of dead weevils was at least 0.99 for weevils sprayed with these products in direct experiments. Previous research has also documented variability in the effect of pyrethrins on insect mortality. Dively et al. [38] found that pyrethrins were able to effectively control pests such as flea beetles (Coleoptera: Chrysomelidae), green peach aphid, *Aphis persicae* Sulzer (Hemiptera: Aphididae), and potato leafhopper, *Empoasca fabae* Harris (Hemiptera: Cicadellidae), but were generally unable to provide sufficient control for the majority of 12 pest groups designated as “difficult-to-control” in organic production, including alfalfa weevil, *Hypera postica* Gyllenhal (Coleoptera: Curculionidae). Spinetoram plus sulfoxaflor has been shown to provide effective control of ornamental and/or nursery crop pests, as this chemical combination significantly reduced the number of male crapemyrtle bark scale, *Acanthococcus lagerstroemiae* Kuwana (Hemiptera: Eriococcidae) pupae (when compared with untreated controls) in crapemyrtle, *Lagerstroemia indica* L., [39] and provided effective control of sweetpotato whitefly, *Bemisia tabaci* Gennadius (Hemiptera: Aleyrodidae) nymphs in poinsettias, *Euphorbia pulcherrima* Willd. ex Klotzsch [40]. In the same study, *B. tabaci* nymphs were also effectively controlled with foliar applications of spirotetramat [40]. Similarly, spirotetramat was found to be toxic to HBW males and females in laboratory toxicity experiments, as <45 µg per weevil caused 50% mortality in both sexes, while 99% mortality was predicted to occur with an application of <230 µg per weevil. While the results from the toxicity experiments differ from the lack of HBW mortality associated with spirotetramat application in other experiments performed in this study, spirotetramat was originally developed for use in the management of difficult-to-control sucking pests [41]. Due to its mode of action as a lipid biosynthesis inhibitor [42], the effect of spirotetramat is particularly pronounced in immature stages of sucking pests [41]. Similar to our results, however, a significant reduction in the reproductive ability of spirotetramat-treated insects has been reported for piercing-sucking pests including *A. persicae* [43], *B. tabaci* [44], and western flower thrips, *Frankliniella occidentalis* Pergande (Thysanoptera: Thripidae) [45].

Our results suggest that the feeding and/or oviposition patterns of the HBW differ across time, as significantly more feeding/oviposition holes were found in hibiscus buds removed at 24 h PES than at any other checkpoint in laboratory experiments, while hibiscus buds collected at 28 days PES contained fewer eggs than at any other PES checkpoint in greenhouse experiments. These findings will likely influence how the HBW is managed, as previous research has found that oviposition patterns can influence pest management tactics. In the congeneric cotton boll weevil, females exhibiting a prolonged ovipositional period in cotton result in staggered adult eclosion from cotton bolls. As a result, sequential insecticide applications are required to control the numerous waves of teneral adults that emerge [12,46,47]. Other biological attributes of the HBW, such as a short development period and thigmotactic behavior, may also complicate its management. In south Florida, propitious environmental conditions (e.g., 6 months with average temperatures exceeding 25 °C) [48] coupled with an egg-to-adult development period of 16 days at 27 °C [5] may favor the occurrence of multiple HBW generations per year. This may further compound the threat of insecticide resistance in the HBW, as pests that have many generations per year have a higher potential for the development of resistance than pests that have one or fewer generations per year [13]. Furthermore, exposure of HBW adults to sources of mortality, including insecticides, may be reduced due to the thigmotactic behavior that these pests exhibit on hibiscus plants [47,49,50]; they can frequently be observed in the area between the flower bud and the bracts (Greene personal observation). Although such behavior may partially limit insecticide efficacy, the identification of insecticide and horticultural oil products capable of causing significant lethal or sublethal effects on HBW adults is still likely to be critical to the successful management of this pest given that the egg, larval, and pupal HBW life stages are found only within developing hibiscus flower buds, which likely provide at least partial protection from insecticides [12].

Significant sublethal or lethal effects were not observed for horticultural oil products (mineral oil (suffOil-X), mineral oil (ultra-fine), paraffinic oil, and thyme and rosemary oil) in this study, with the exception of the results from direct spray experiments, as each product caused 100% HBW mortality in each replicate by the first PES checkpoint (24 h). These results match expectations, as horticultural oils must be applied directly to the arthropod pest, given that their residual activity is low [17]. Horticultural oils are most effective on lightly sclerotized arthropods, such as scales, aphids, whiteflies, thrips, and mealybugs [17], and may not always be effective on hard-bodied insects such as coleopterans. Similar to our results, however, significant mortality in congeneric *A. eugenii* adults occurred when they were treated with mineral oil [51]. Because pests must come into direct contact with horticultural oil products for them to be effective, these products may not be the first choice for HBW management due to the additional protection associated with the thigmotactic behavior of HBW adults on hibiscus, in addition to the fact that the immature stages are concealed within flower buds. However, the inclusion and rotation of horticultural oils into an IPM program can provide several advantages over conventional insecticides, including a lower risk of soil and groundwater contamination, resistance development, and negative effects on natural enemies [17,52].

As we seek to progress towards the development of an IPM program for the HBW, the identification of efficacious insecticide products belonging to different IRAC groups is important for resistance management and the sustainability of the hibiscus industry in south Florida. Diflubenzuron (group 15) and pyrethrins (group 3A) are of particular interest given their designation as reduced-risk pesticides [53,54], while the combined effect of the two distinct IRAC groups in spinetoram plus sulfoxaflor (groups 4C and 5) resulted in significant mortality and a reduction in the reproductive rate of the HBW. While spirotetramat significantly altered HBW feeding and oviposition behavior when applied as a contact insecticide in this study, its ability to translocate throughout the entirety of a plant’s vascular system as a systemic insecticide can also provide plant protection from a number of different pests, including aphids, mealybugs, psyllids, scales, and whiteflies [41]. Because hibiscus nurseries in Florida must contend with a menagerie of pests in addition to the HBW, the identification of versatile insecticide and horticultural oil products that can be applied in different ways or used in the management of various pests is an important finding of this study. Cotton stainers, *Dysdercus suturellus* (Herrich-Schaeffer) (Hemiptera: Pyrrhocoridae) [55], lobate lac scales, *Paratachardina lobata lobata* (Chamberlin) (Hemiptera: Kerriidae) [56], cotton aphids, *Aphis gossypii* Glover (Hemiptera: Aphididae) [57], papaya mealybugs, *Paracoccus marginatus* Williams and Granara de Willink (Hemiptera: Pseudococcide) [58], pink hibiscus mealybugs, *Maconellicoccus hirsutu* (Green) (Hemiptera: Pseudococcide) [59], hibiscus bud midges, *Contarinia maculipennis* Felt (Diptera: Cecidomyiidae) [60], and two-spotted spider mites, *Tetranychus urticae* Koch (Acari: Tetranychidae) [61], have all been detected on hibiscus in Florida. To better manage the HBW and the complex of pests that attack hibiscus, it is our recommendation that foliar applications of diflubenzuron, pyrethrins, spinetoram plus sulfoxaflor, and spirotetramat be rotated alongside horticultural oils (e.g., mineral, paraffinic, and thyme and rosemary oil) in south Florida nurseries.

Our results constitute an important first step in the identification of chemical control options for the HBW in hibiscus, but it will be important to monitor the efficacy of these products as they are applied on a commercial scale and to continue to identify additional chemical, biological, cultural, and mechanical control options to effectively manage the complex of pests that attack hibiscus nurseries in Florida. Although immature HBW stages developing within hibiscus buds may not be highly susceptible to the contact insecticides identified in this study, future research may allow us to better manage these life stages through management options such as systemic insecticide application or biological control. Implementation of additional management practices such as sanitation (e.g., removing fallen hibiscus buds) may also be able to reduce HBW numbers through the prevention of generational buildup following initial colonization events. Furthermore, a better understanding of the spatiotemporal distribution of the HBW within nurseries and surrounding agroecosystems may allow for sources of infestation (e.g., non-crop host plants) to be identified and for areas of high and low pest pressure to be delineated so that management efforts may be applied in the areas that need it most or in particularly critical times for pest dispersion, such as the shipping season. In summary, this study presents a comprehensive screening of the effect of 21 different insecticide and horticultural oil products (12 distinct IRAC modes of action) on a newly invasive pest of hibiscus, the hibiscus bud weevil. In addition to providing information on managing current infestations, baseline knowledge of the effect of readily accessible insecticides and horticultural oils on HBW mortality and biology will be useful in future management scenarios, such as the selection of products to work in combination with biological control or to guide insecticide resistance management for this portentous pest of hibiscus.

## Figures and Tables

**Table 1:** Insecticide and horticultural oil products selected for testing in laboratory and greenhouse experiments.

	Trade Name	Active Ingredient(s)	Insecticide Group	Rate ^a^	Solution Prepared
Insecticides	Acelepryn	Chlorantraniliprole	28	1.2 L/ha	1.25 mL/L
	Acephate 97UP WDG	Acephate	1B	840 g/ha	898 mg/L
	AzaSol	Azadirachtin	Unknown	420 g/ha	899 mg/L
	Conserve SC	Spinosad	5	1.8 L/ha	0.468 mL/L
	Decathlon 20 WP	Cyfluthrin	3A	133 g/ha ^b^	143 mg/L
	Dimilin 25 W	Diflubenzuron	15	1121 g/ha	1.2 g/L
	Hachi-Hachi SC	Tolfenpyrad	21A		2.11 mL/L
	Imidan 70-W	Phosmet	1B	1121 g/ha	1.2 g/L
	Kontos	Spirotetramat	23	0.25 L/ha	0.264 mL/L
	Mainspring GNL	Cyantraniliprole	28	0.59 L/ha ^b^	0.62 mL/L
	Mavrik Aquaflow	Tau-fluvalinate	3A	1.6 L/ha	0.78 mL/L
	PyGanic Crop Protection EC 5.0 II	Pyrethrins	3A	1.1 L/ha	1.22 mL/L
	Scimitar GC	Lambda-Cyhalothrin	3A	0.37 L/ha ^b^	0.39 mL/L
	Sevin SL	Carbaryl	1A	2.3 L/ha	2.5 mL/L
	Talstar P	Bifenthrin	3A	1.6 L/ha	1.7 mL/L
	Timectin 0.15 EC T&O	Abamectin	6	1.2 L/ha	0.2 mL/L
	Xxpire	Spinetoram plus Sulfoxaflor	4C and 5	385 g/ha	203 mg/L
Horticultural oils	Agropest	Thyme and Rosemary Oil	Unclassified	4.7 L/ha ^c^	5 mL/L
	JMS Stylet-Oil	Paraffinic Oil	Unclassified	7.3 L/ha ^b^	7.8 mL/L
	SuffOil-X	Mineral Oil	Unclassified	18.7 L/ha ^b^	20 mL/L
	Ultra-fine	Mineral Oil	Unclassified	47 L/ha	20 mL/L

^a^ Unless otherwise noted, rate calculations are based on the label-recommended amount of product to be applied to one hectare. ^b^ Because the manufacturer label did not include recommendations for area, the rate was calculated assuming an application rate of 935 L/ha. ^c^ Because label recommendations are in *v*/*v* for this product, the rate was calculated as a 0.5% solution assuming an application rate of 935 L/ha.

**Table 2:** Median lethal dose (LD_50_; probit analysis) comparison between hibiscus bud weevil (HBW) males and females (*t*-tests) for selected insecticides.

Insecticide	Sex	*n* ^a^	Slope ^b^ ± SE	LD_50_, µg/Weevil (95% CI)	χ^2^	df	*p*-Value
Diflubenzuron ^c^	♂	50	-	-	-	-	-
	♀	50	-	-	-	-	-
Spinetoram plus Sulfoxaflor	♂	145	3.89 ± 0.34	0.06 (0.05–0.07)	1.55	2	0.46
	♀	147	3.74 ± 0.33	0.06 (0.06–0.07)	2.26	2	0.32
Pyrethrins	♂	180	1.00 ± 0.11	0.07 (0.03–0.16)	6.30	3	0.10
	♀	180	1.01 ± 0.11	0.10 (0.04–0.23)	6.88	3	0.08
Spirotetramat	♂	175	3.58 ± 0.29	41.72 (37.63–45.98)	3.58	2	0.17
	♀	152	3.26 ± 0.27	44.36 (39.81–49.16)	3.92	2	0.14

^a^ Total number of HBW adults tested. ^b^ Coefficient of the regression of log (dose) on HBW mortality used to calculate the LD_50_. ^c^ Because adult mortality was not observed 24 h after treatment at the max label rate, a probit analysis was not conducted for diflubenzuron.

## Data Availability

The data presented in this study will be available at https://doi.org/10.6084/m9.figshare.22942379 upon acceptance of the manuscript.

## Supplementary Materials

The following supporting information can be downloaded at: https://www.mdpi.com/article/10.3390/insects14060544/s1, Supplementary Figure S1: Generalized linear mixed model (GLMM)-predicted proportion of HBW mortality (±SE) and observed HBW mortality (±SE) among insecticide and horticultural oil products from hibiscus bud fixed experiments; Supplementary Figure S2: Generalized linear mixed model (GLMM)-predicted proportion of HBW mortality (±SE) and observed HBW mortality (±SE) among insecticide and horticultural oil products from hibiscus leaf fixed experiments; Supplementary Figure S3: Generalized linear mixed model (GLMM)-predicted proportion of HBW mortality (±SE) and observed HBW mortality (±SE) among insecticide and horticultural oil products from direct experiments; Supplementary Figure S4: Generalized linear mixed model (GLMM)-predicted proportion of HBW mortality (±SE) and observed HBW mortality (±SE) among insecticide and horticultural oil products from hibiscus bud replacement experiments; Supplementary Figure S5: Generalized linear mixed model (GLMM)-predicted mean number (±SE) of HBW eggs among insecticide and horticultural oil product treatments from hibiscus bud replacement experiments; Supplementary Figure S6: Generalized linear mixed model (GLMM)-predicted mean number (±SE) of HBW feeding/oviposition holes among insecticide and horticultural oil product treatments from hibiscus bud replacement experiments; Supplementary Figure S7: Generalized linear mixed model (GLMM)-predicted mean number (±SE) of HBW eggs among insecticide product treatments from hibiscus bud greenhouse experiments; Supplementary Figure S8: Generalized linear mixed model (GLMM)-predicted mean number (±SE) of HBW feeding/oviposition holes among insecticide product treatments from hibiscus bud greenhouse experiments; Supplementary Figure S9: Generalized linear mixed model (GLMM)-predicted mean number (±SE) of HBW larvae among insecticide product treatments from hibiscus bud greenhouse experiments.
