# Supplementary material for: Lethal and Sublethal Effects of Contact Insecticides and Horticultural Oils on the Hibiscus Bud Weevil, Anthonomus testaceosquamosus Linell (Coleoptera: Curculionidae)
Source: Insects. 2023 Jun 11;14(6):544. doi: 10.3390/insects14060544 (PMC10299327; doi:10.3390/insects14060544)
Supplement: Supplementary file 1 [file insects-14-00544-s001.zip › insects-2433668-supplementary.pdf]

Lethal and Sublethal Effects of Contact Insecticides and Horticultural Oils on the Hibiscus Bud Weevil, *Anthonomus testaceosquamosus* Linell (Coleoptera: Curculionidae)

A. Daniel Greene[1,3] (ORCID: 0000-0002-0877-2377), Xiangbing Yang[2] (ORCID: 0000-0001-7072-5515), Yisell Velazquez-Hernandez[1], German Vargas[1] (ORCID: 0000-0002-8273-0140), Paul E. Kendra[2] (ORCID: 0000-0003-4425-0733), Catharine Mannion[1] and Alexandra M. Revynthi[1,3] (ORCID: 0000-0002-3284-3104)

[1]Entomology and Nematology Department, Tropical Research and Education Center, University of Florida, 18905 SW 280th St, Homestead, FL 33031, USA

[2] Subtropical Horticulture Research Station, United States Department of Agriculture, Agricultural Research Service, Miami, FL 33158, USA

[3] Corresponding authors; anthonygreene@ufl.edu and arevynthi@ufl.edu

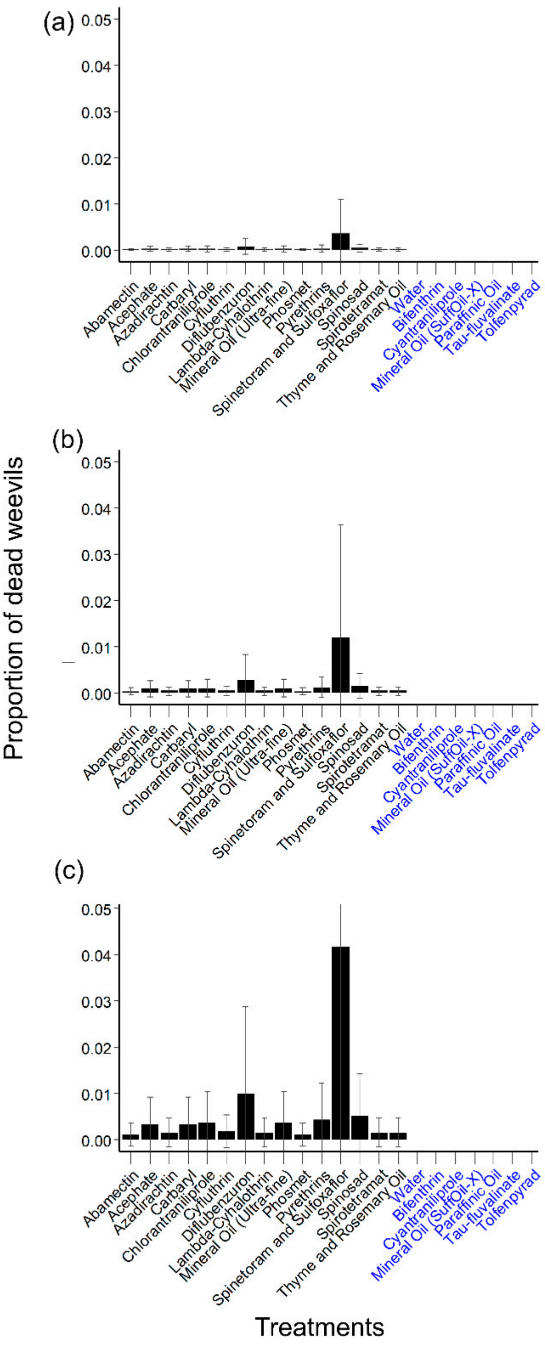

Supplementary Figure S1 Generalized linear mixed model (GLMM)-predicted proportion of HBW mortality (± SE) and observed HBW mortality (± SE) among insecticide and horticultural oil products from hibiscus bud fixed experiments. (a) 48 hours post-experimental setup (PES), (b) 72 hours PES, and (c) 96 hours PES. Black = modeled treatments; GLMM-predicted proportion of mortality. Blue = treatments not included in models due to causing 0 or 100 percent mortality (model convergence); observed mortality data. Mortality was not observed during the 24 hours PES checkpoint. Treatments with different letters were significantly different from one another ($p < 0.05$; t-tests)

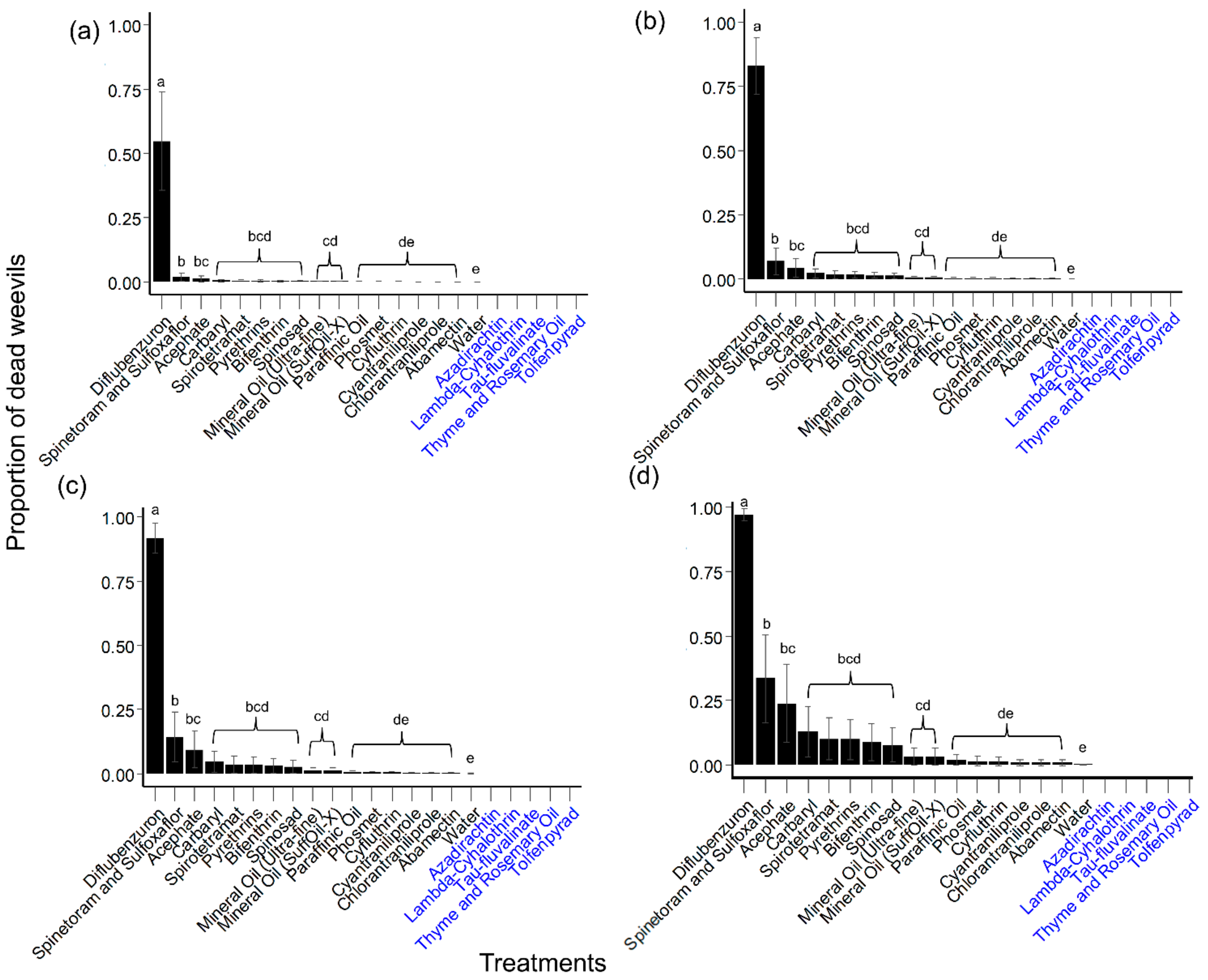

Supplementary Figure S2 Generalized linear mixed model (GLMM)-predicted proportion of HBW mortality (± SE) and observed HBW mortality (± SE) among insecticide and horticultural oil products from hibiscus leaf fixed experiments. (a) 24 hours post-experimental setup (PES), (b) 48 hours PES, (c) 72 hours PES, and (d) 96 hours PES. Black = modeled treatments; GLMM-predicted proportion of mortality. Blue = treatments not included in models due to causing 0 or 100 percent mortality (model convergence); observed mortality data. Treatments with different letters were significantly different from one another ($p < 0.05$; t-tests)

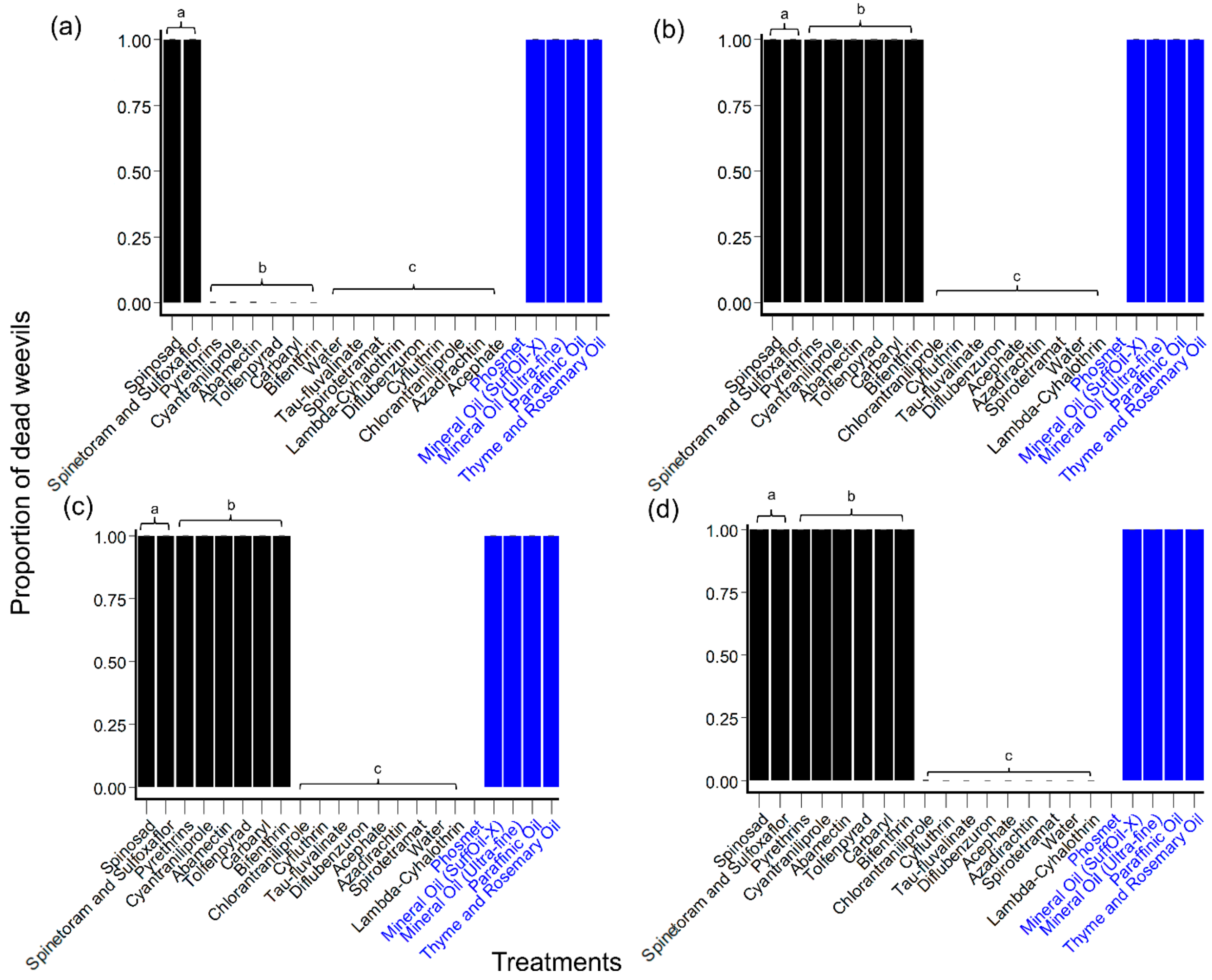

Supplementary Figure S3 Generalized linear mixed model (GLMM)-predicted proportion of HBW mortality (± SE) and observed HBW mortality (± SE) among insecticide and horticultural oil products from direct experiments. (a) 24 hours post-experimental setup (PES), (b) 48 hours PES, (c) 72 hours PES, and (d) 96 hours PES. Black = modeled treatments; GLMM-predicted proportion of mortality. Blue = treatments not included in models due to causing 0 or 100 percent mortality (model convergence); observed mortality data. Treatments with different letters were significantly different from one another ($p < 0.05$; t-tests)

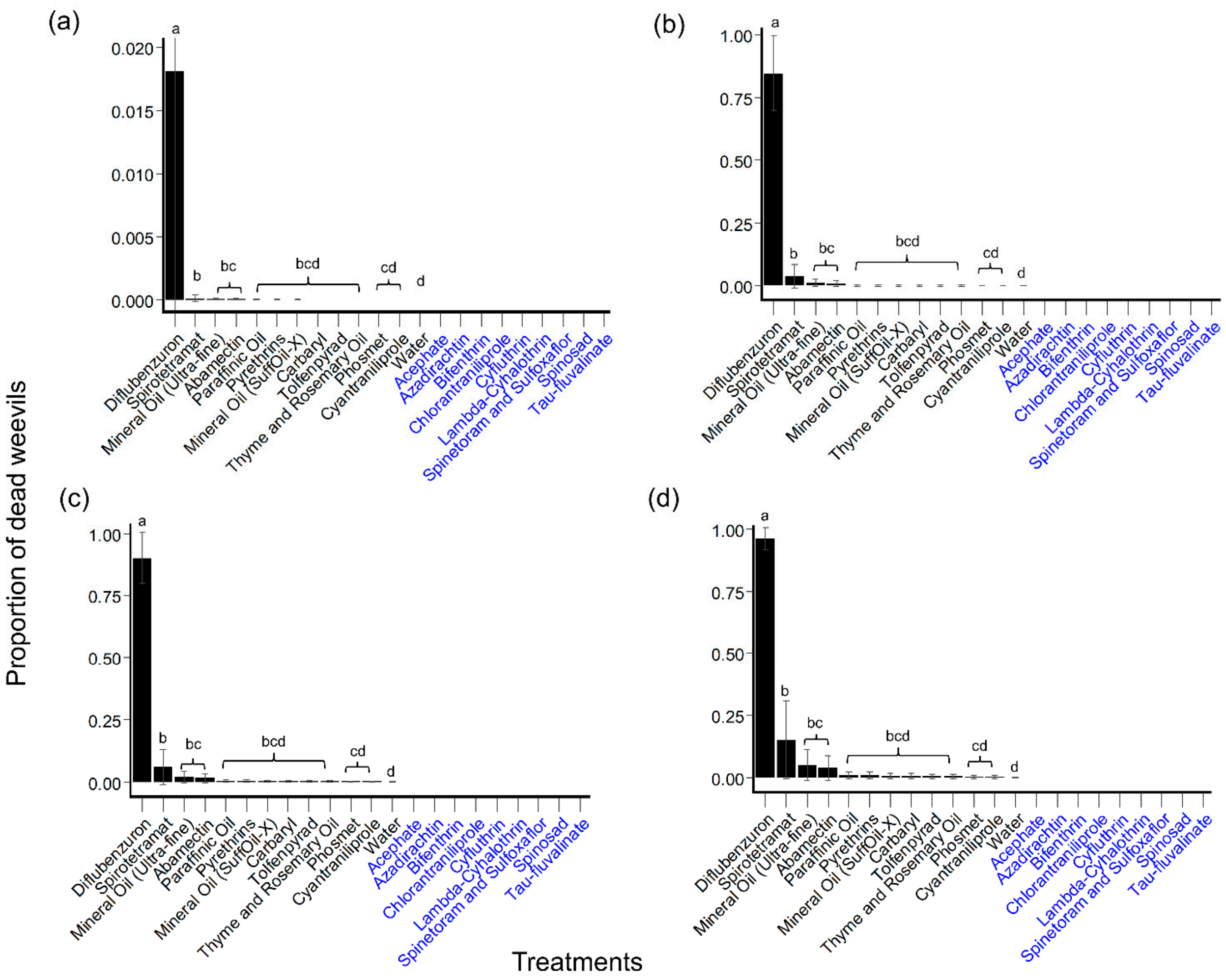

Supplementary Figure S4 Generalized linear mixed model (GLMM)-predicted proportion of HBW mortality (± SE) and observed HBW mortality (± SE) among insecticide and horticultural oil products from hibiscus bud replacement experiments. (a) 24 hours post-experimental setup (PES), (b) 48 hours PES, (c) 72 hours PES, and (d) 96 hours PES. Black = modeled treatments; GLMM-predicted proportion of mortality. Blue = treatments not included in models due to causing 0 or 100 percent mortality (model convergence); observed mortality data. Treatments with different letters were significantly different from one another ($p < 0.05$; t-tests)

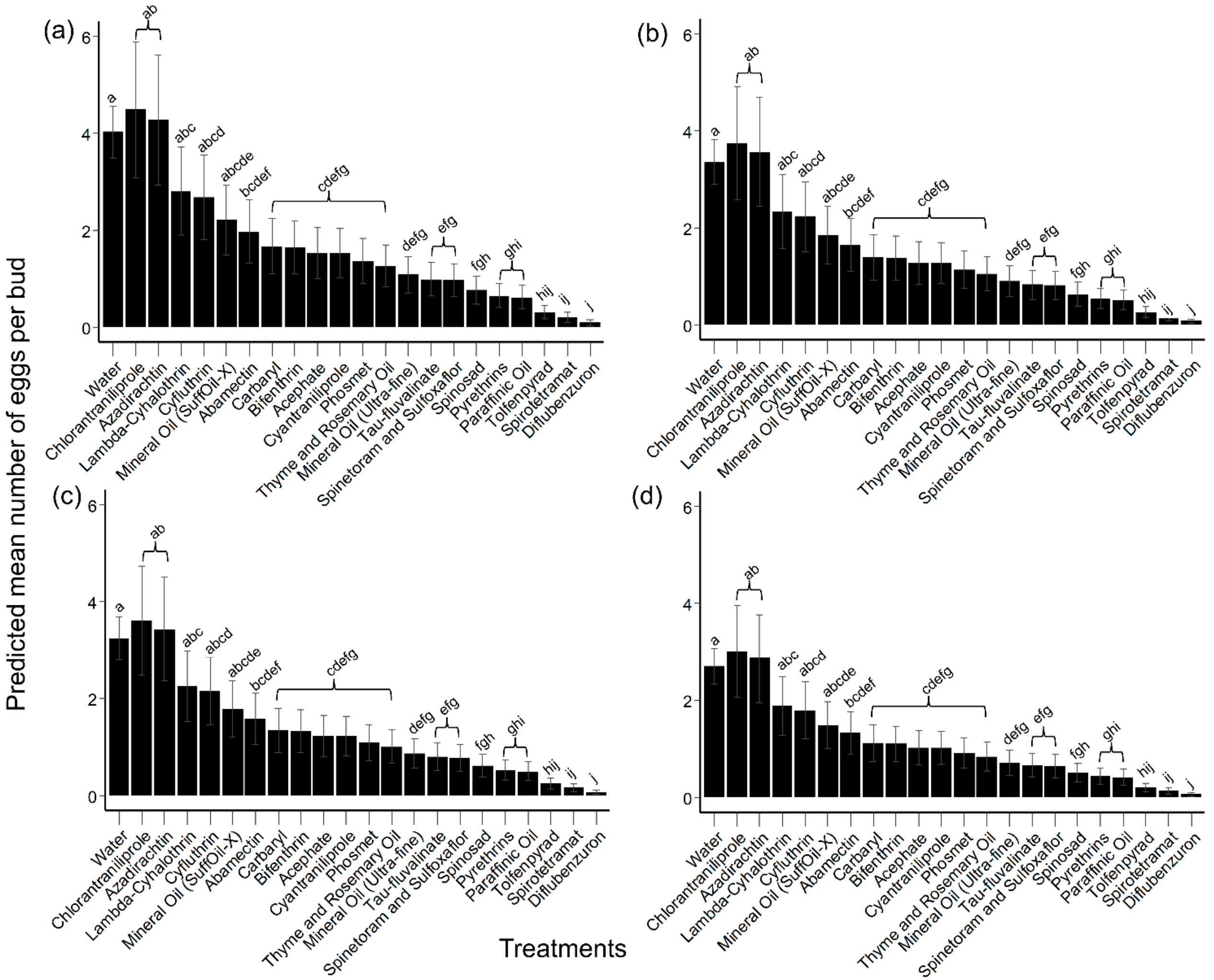

Supplementary Figure S5 Generalized linear mixed model (GLMM)-predicted mean number (± SE) of HBW eggs among insecticide and horticultural oil product treatments from hibiscus bud replacement experiments. (a) 24 hours post-experimental setup (PES), (b) 48 hours PES, (c) 72 hours PES, and (d) 96 hours PES. Treatments with different letters were significantly different from one another ($p < 0.05$; t-tests)

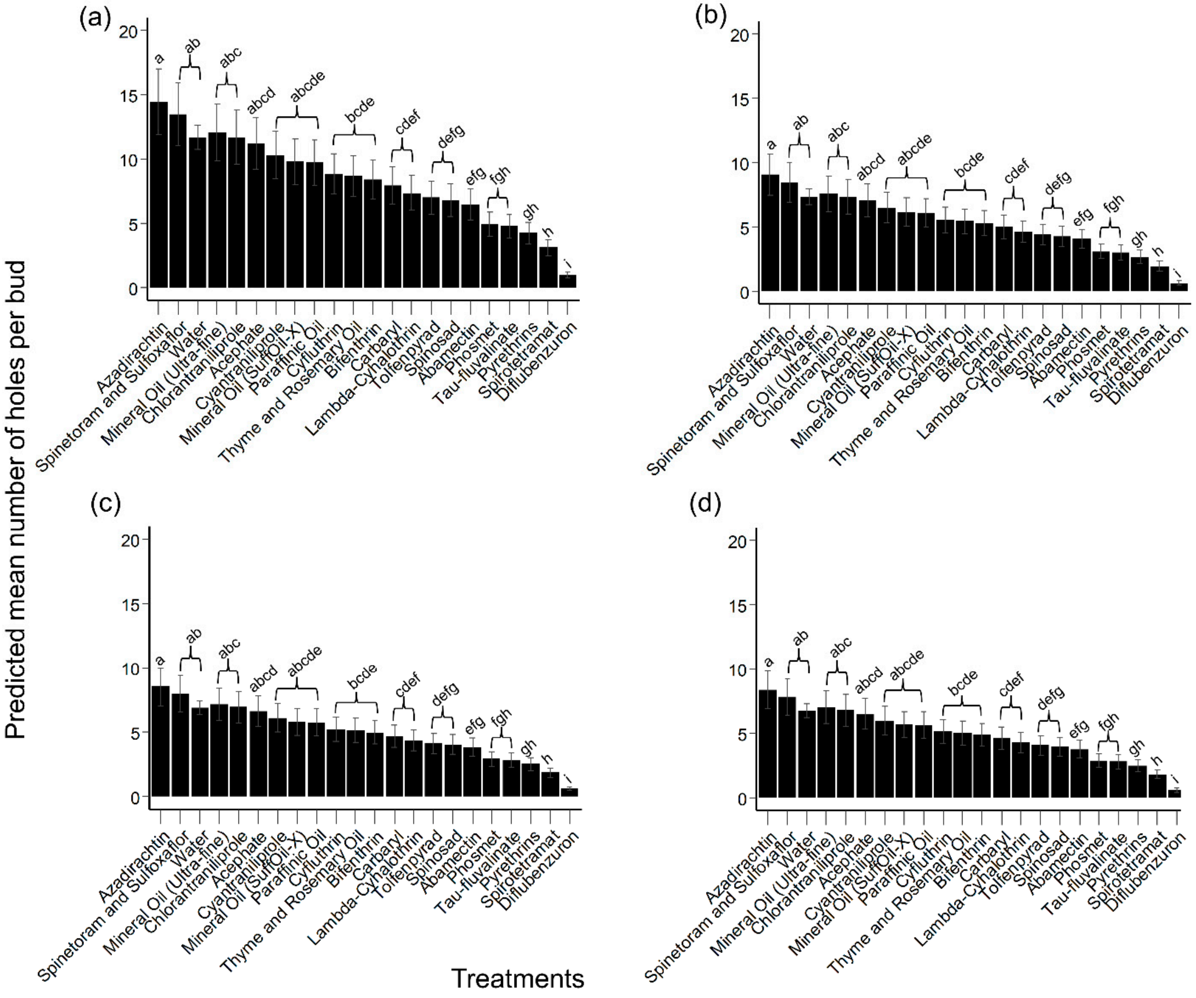

Supplementary Figure S6 Generalized linear mixed model (GLMM)-predicted mean number (± SE) of HBW feeding/oviposition holes among insecticide and horticultural oil product treatments from hibiscus bud replacement experiments. (a) 24 hours post-experimental setup (PES), (b) 48 hours PES, (c) 72 hours PES, and (d) 96 hours PES. Treatments with different letters were significantly different from one another ($p < 0.05$; t-tests)

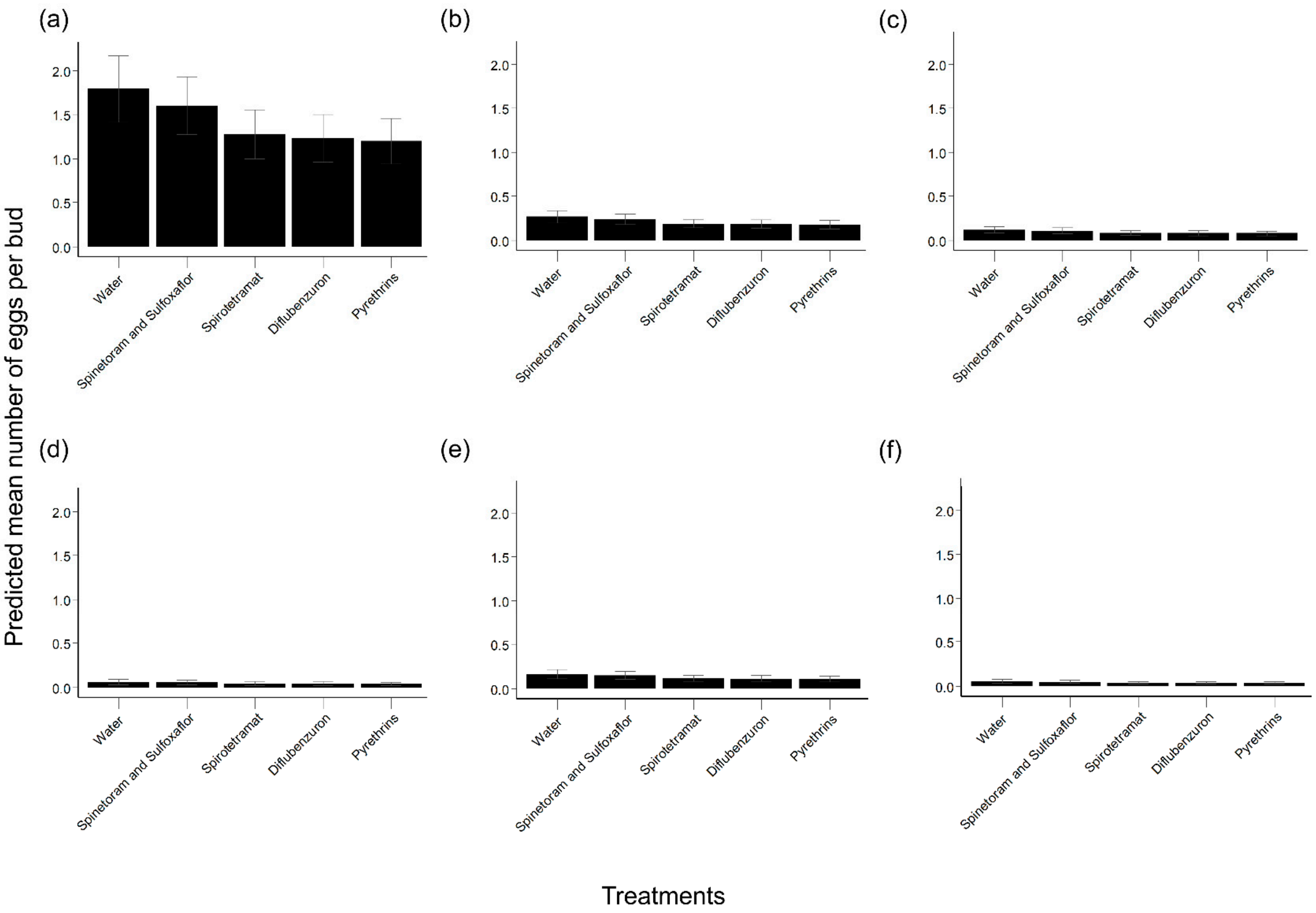

Supplementary Figure S7 Generalized linear mixed model (GLMM)-predicted mean number (± SE) of HBW eggs among insecticide product treatments from hibiscus bud greenhouse experiments. (a) Prior to treatment application, (b) 1 day post-experimental setup (PES), (c) 4 days PES, (d) 7 days PES, (e) 19 days PES, and (f) 28 days PES

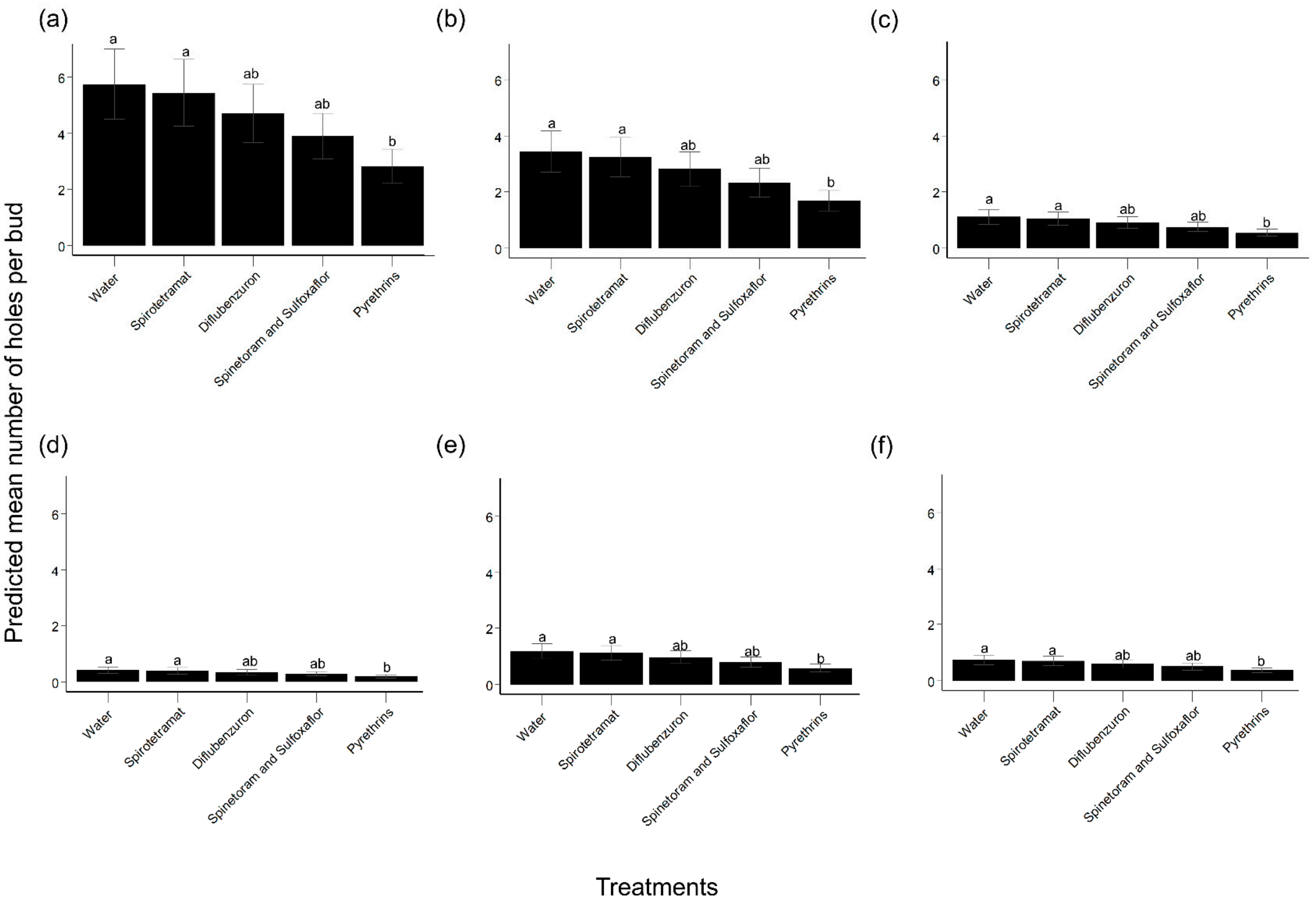

Supplementary Figure S8 Generalized linear mixed model (GLMM)-predicted mean number (± SE) of HBW feeding/oviposition holes among insecticide product treatments from hibiscus bud greenhouse experiments. (a) Prior to treatment application, (b) 1 day post-experimental setup (PES), (c) 4 days PES, (d) 7 days PES, (e) 19 days PES, and (f) 28 days PES. Treatments with different letters were significantly different from one another ($p < 0.05$; t-tests)

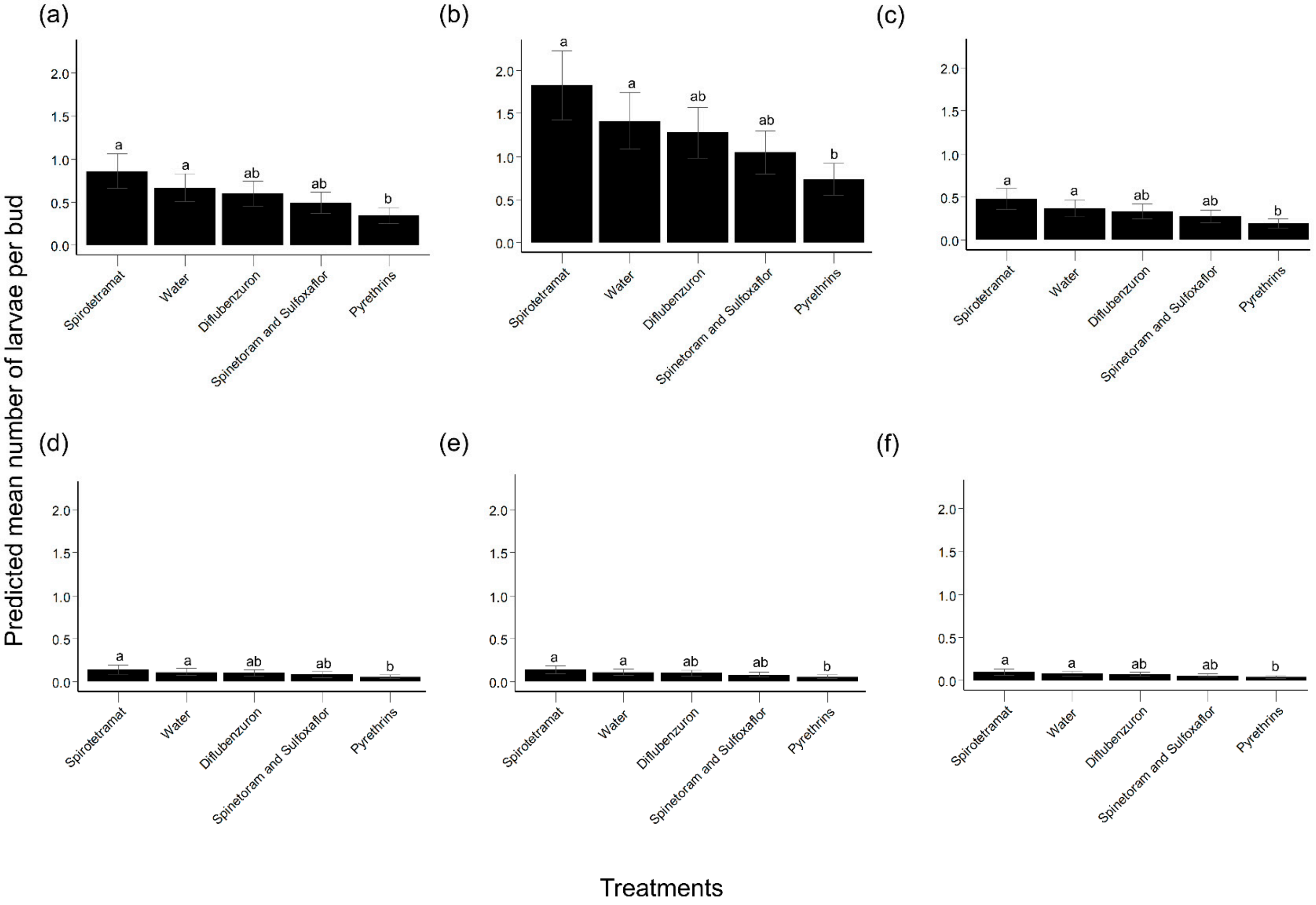

Supplementary Figure S9 Generalized linear mixed model (GLMM)-predicted mean number (± SE) of HBW larvae among insecticide product treatments from hibiscus bud greenhouse experiments. (a) Prior to treatment application, (b) 1 day post-experimental setup (PES), (c) 4 days PES, (d) 7 days PES, (e) 19 days PES, and (f) 28 days PES. Treatments with different letters were significantly different from one another ($p < 0.05$; t-tests)
